# Facilitators and barriers of sociodemographic data collection in Canadian health care settings: a multisite case study evaluation

**DOI:** 10.1186/s12939-018-0903-0

**Published:** 2018-12-27

**Authors:** Hazel Williams-Roberts, Cory Neudorf, Sylvia Abonyi, Jennifer Cushon, Nazeem Muhajarine

**Affiliations:** 1 Department of Community Health and Epidemiology, University of Saskatchewan, Saskatoon, Canada; 2 Population and Public Health, Saskatchewan Health Authority, Saskatoon, Canada; 3 Saskatchewan Population Health and Evaluation Unit (SPHERU), Saskatoon, Canada

**Keywords:** Facilitators, Barriers, Implementation research, Social determinants of health, Health services

## Abstract

**Background:**

Despite growing awareness of the importance of social determinants of health, research remains limited about the implementation of sociodemographic data collection in Canadian health care settings. Little is known about the salient contextual factors that enable or hinder collection and use of social information to improve quality of care in clinical settings. This study examines the perceptions and experiences of managers and care providers to better understand how to support organizational efforts to collect and use sociodemographic data to provide equity-oriented care.

**Methods:**

Case studies of three diverse urban health care settings employed semi-structured individual and group interviews with managers and care providers respectively to explore their experiences with implementation. Data was analyzed separately and in context for each site as part of an individual case study. A thematic analysis of interview transcripts was performed with an inductive approach to coding of segments of the text. Constructs of the Consolidated Framework for Implementation Research (CFIR) were used as an analytical framework to structure the data to support cross case comparisons of facilitators and barriers to implementation across settings.

**Results:**

Several perceived facilitators and barriers to implementation were identified that clustered around three CFIR domains: intervention, inner setting and characteristics of individuals. Macro level (outer setting) factors were relatively unexplored. Sites were motivated by their recognition of need for social information to improve quality of care. Organizational readiness for implementation was demonstrated by priorities that reflected concern for equity in care, leadership support and commitment to an inclusive process for stakeholder engagement. Barriers included perceived relevance of only a subset of sociodemographic questions to service delivery, staff capacity and comfort with data collection as well as adequate resources (funding and time).

**Conclusion:**

Although system level mandates were underexplored, they may accelerate adoption and implementation of sociodemographic data collection in the presence of organizational readiness. Standardized tools integrated into information systems and workflows would support adequately trained personnel. More research is needed to understand important factors in rural health settings and with clinical application to inform care delivery pathways.

**Electronic supplementary material:**

The online version of this article (10.1186/s12939-018-0903-0) contains supplementary material, which is available to authorized users.

## Introduction

There is increasing global recognition that conditions of daily living affect opportunities for healthy choices [1, 2]. Despite this awareness, Canadian health care systems have not consistently captured or applied important information on individual patient social factors [3]. Historically in Canada, only limited information such as age, sex and residence have been collected; although other factors including ethnicity, language and sexual orientation influence outcomes, care experiences and satisfaction [2]. The paucity of social information creates missed opportunities to understand the social needs of the population served by health care facilities, identify health disparities and tailor individual care to address social barriers [4]. To change the status quo, more research is needed about how to collect and apply information about social factors in culturally appropriate and acceptable ways.

There is limited and mixed experience with implementation of sociodemographic data collection in Canadian health care settings [5–9]. Additionally, a few studies in larger metropolitan centres suggest that regional variation exists in public support for sociodemographic data collection, as well as a gradient of comfort depending on the question [10, 11]. This advances a compelling case for implementation research to better understand local concerns, preferences and develop effective strategies to overcome challenges.

The collection of sociodemographic information has multiple and varied uses in health services. This includes tailoring individual care to address health-related social barriers, aggregation of individual data to understand the social needs of persons who access services and inform health system planning and resource allocation, and identification of health inequalities in processes and outcomes [4]. A nascent but growing of body of research has focused on intervention at the individual level and several social screening questions/tools have explored a heterogeneous group of social domains, mostly in US pediatric primary care settings [12–19]. However, few tools have been rigorously evaluated and contextual factors are likely to influence feasibility and acceptability of implementation in different settings [12]. A better understanding of factors influencing implementation will pave the way for introduction and evaluation of standardized tools that are well suited to programs.

The limited availability of sociodemographic data to support measurement of health equity as an indicator of health system performance has also been recognized by the Canadian Institute for Health Information (CIHI) [20]. Stakeholders have been mobilized to identify core sociodemographic stratifiers to be used to measure health inequalities in the near future. The study supports the national efforts and aims to understand the salient factors that are necessary to support health care systems to introduce these upstream and equity-oriented approaches to care delivery.

## Methods

### Design

This paper discusses a component of a larger multiple case study evaluation that assessed the feasibility and acceptability of collection and use of selected sociodemographic data in three diverse urban health settings of a medium sized city in a western prairie province. A “case” referred to a unit (i.e. a program, department or entire organization), that implemented sociodemographic data collection in a given site. There was an instrumental intent in the use of collective case studies [21]. Different sites reflected variation in context, target populations and processes of implementation of sociodemographic data collection. All existing cases were studied. Within cases, the study focused on the perceptions and experiences of various groups of participants who were either providers of services or key decision makers who presided over administration of the project [22].

The researchers had a broad vision that included implementation of sociodemographic data collection and its application at the point of care.. However, it was impractical to impose standardized data collection as a condition for case participation. During discussions with implementation teams, it was necessary to be flexible and to allow for adaptation of implementation to their contexts, resources, realities, and prioritization of their information needs.

### Setting

The three cases offered unique contexts for implementation of sociodemographic data collection. Case 1 described implementation in a publicly-owned and operated primary care centre that offered the full complement of primary health services including maternal and child health, oral health, home visiting, chronic disease management, and health promotion. The changing demographic and cultural mosaicism of the catchment area served by the centre prompted the manager of the centre to want an examination of clients’ service needs and preferences. So the manager requested support to conduct a survey among immunization clients. This serendipitous opportunity allowed the researchers to include sociodemographic questions to provide a context for service-related results. Although the information was not integrated at the point of care, the manager intended the survey results to inform service planning and assist management to deliver immunization services in a more responsive way that meets community needs and preferences.

Case 2 described implementation of sociodemographic data collection in a community-based organization that provided sexual and reproductive health services to an underserved population. Funding for the centre’s operations is obtained through grants, fund raising events and donations. Increasingly, potential municipal donors to the centre were requesting additional information to better characterize the needs of clients who access services. The centre instituted expanded sociodemographic data collection for new clients as part of the usual intake assessment. The information was applied to tailor care and treatment, as well as better understand the characteristics of the population being served.

Case 3 described implementation of sociodemographic data in the context of a hospital registration department. The facility is surrounded by several communities that are among the most socioeconomically deprived in the city. There is also a higher concentration of Indigenous peoples who reside in these communities. The implementation of data collection related to Indigenous identity supported the delivery of cultural support and navigation services. Before the study was conducted, the hospital relied on surname and home community analysis of the daily census to identify Indigenous patients. Cultural support services were then offered to these individuals in an effort to provide culturally appropriate care. The limitations and opportunities for misclassification with this method were recognized. The hospital thought that facilitating patients to self-identify as Indigenous at registration would increase the yield, preserve patient right to choose whether they disclosed their identity, and provide valuable information to support program planning.

### Participants

Study participants included managers and health care providers. In each case, key informants were middle managers; however, in one site a key informant was the executive director because of the organization’s structure. All key informants were in positions of authority, knowledgeable about the institution’s policies, operations, culture, and implementation procedures. They also influenced key decisions about implementation of sociodemographic data collection in each of the respective sites.

The types of health care providers involved in implementation varied across cases. Case 1 included 12 nurse providers who delivered immunization and child wellness services. In Case 2, a four-member interdisciplinary team included a nurse practitioner, social worker, counsellor and support staff. In Case 3, no clinical care providers were directly involved in sociodemographic data collection due to the structure of the organization. All participants were female adults reflecting the gendered nature of the health care workforce [23].

### Data collection

#### Sociodemographic data collection

A robust process was developed to identify sociodemographic questions that were appropriate for a broad array of care settings. Two criteria for identification of candidate sociodemographic questions included: 1) evidence of existence of health disparities related to a particular sociodemographic domain and, 2) feasibility of collection of a particular sociodemographic question. Feasibility was assessed by a combination of factors including prior experience with collection in other settings (e.g. Census, Population health surveys), availability of similar information from alternate sources, potential sensitivity, and client willingness to disclose the information. Where available, validated sociodemographic questions were preferred. Once a set of candidate questions had been identified, the researchers embarked on a process of consultation with health care managers as well as community-based organizations that work with populations that experience vulnerabilities (e.g. immigrants and refugees, sexual and gender minorities, persons living with disabilities, Indigenous peoples). The final list of sociodemographic questions can be found in Additional file 1.

Cases varied in the number and mode of administration of sociodemographic questions. The researchers worked to integrate sociodemographic data collection into the existing work flow. Two cases chose to employ self-administered paper questionnaires as their process. This offered the advantage of privacy, potentially increasing patient comfort, when responding to sensitive questions. However, this modality also required that patients have the ability to read and understand the questions. Questionnaires were only available in English and staff provided support to patients who requested assistance with completing the questionnaire.

Case 3 chose to only collect information about Indigenous identity at registration. This question was integrated alongside other questions that registration personnel routinely asked such as date of birth, address, emergency contact person, and family physician.

#### Research-related data collection

Within case studies, qualitative methods provided rich detailed information for understanding the context of implementation as well as elucidating the perceived facilitators and barriers to change in the various settings [24]. The study used individual semi-structured interviews with key informants, informal discussions and focus groups with care providers to examine participants’ perceptions and experiences.

Interviews were structured according to an interview guide with open ended questions that focused conversations on experiences with implementation including enablers, challenges and supports needed to sustain data collection. Questions were not asked specifically about constructs included in CFIR. All participants gave written consent prior to the interview and permission was obtained for audiotaping of interviews. All interviews occurred in a private space at a mutually agreed location.

The informal group discussion was shorter than either focus groups or individual interviews (38–45 min) because it occurred after a routine team huddle as it was difficult to find an alternative time to meet with nurses during work hours. Participants were offered the opportunity to review their interview transcripts. The study received institutional ethics approval from the university’s behavioral ethics review board (BEH# 15–228).

### Analysis

A thematic analysis was conducted of transcripts in each case [25]. All transcripts were printed and read repeatedly to encourage familiarity with content. Segments of texts were labelled with codes in the margins using an open coding process. Codes were clustered into categories based on related ideas in the data. Categories were subsequently mapped to themes using the comprehensive taxonomy of constructs in the Consolidated Framework of Implementation Research (CFIR) as an analytical framework [26]. This was an interpretive process as the coder (HWR) was required to assign a category to a matching CFIR construct based on its content.

The CFIR has five multilevel domains including characteristics of intervention, outer setting (external factors such as policy and incentives), inner setting (organizational factors), characteristics of individuals (e.g. knowledge and beliefs about the intervention) and process of implementation (e.g. engagement of stakeholders) [26]. The use of previously defined and operationalized constructs was practical and allowed for framing of results in a way that enabled comparison with other studies [27].

Upon completion of all three case studies, a cross case description and comparison of factors influencing implementation was conducted. The factors identified in each case were charted in a table (Table 1) to allow for comparisons between cases and identify similarities, differences and patterns. Excerpts of the data have been provided to illustrate themes as reflected by various CFIR constructs.

**Table 1:** Facilitators and barriers to implementation of sociodemographic data collection

Cases	Themes		
	Theme 1: Intervention characteristics		
	Relative advantage	Adaptability	
Case 1	The benefits of sociodemographic data collection were recognized and described by managers in all three cases (+)	The process of implementation was adapted to each context (+)	Only a subset of sociodemographic questions was perceived to be relevant (−)
Case 2			Not mentioned
Case 3			A single question about Indigenous identity was implemented (−)
	Theme 2: Outer setting		
	External policies and incentives		
Case 1	Not mentioned		
Case 2	Required by funders to collect sociodemographic information (+)		
Case 3	Not mentioned		
	Theme 3: Inner setting		
	Relative priority and perceived tension for change	Readiness for change	Availability of resources
Case 1	District review had already prioritized social determinants of health and was consistent with the focus on enhancing sociodemographic data collection (+)	All managers described engaged leadership and support for implementation of sociodemographic data collection (+)	Limited time for clinical tasks (−)
Case 2	Core service priorities were well aligned with implementation of sociodemographic data collection (+)		Added time not perceived as value added for patients (−) Limited staff and finances for implementation (−)
Case 3	Current approaches for targeting Indigenous individuals for cultural support were not optimal (+)		Legacy IT system limited the number of questions and response options that could be added (−)
	Theme 4: Characteristics of individuals		
	Knowledge, attitudes and beliefs about the intervention		
Case 1	Perception that some care providers were uncomfortable with sociodemographic data collection		
Case 2	Staff had experience and were already collecting some sociodemographic data (+)		
Case 3	Manager described staff discomfort with data collection (−)		Staff perceived that patients would be uncomfortable with data collection (−)

(+) = Facilitator to implementation (−) = Barrier to implementation

Signs do not indicate magnitude

Weekly de-briefing sessions with the project team enabled the first author to question assumptions and remain grounded in the data. Preliminary findings were discussed with stakeholders including implementation partners to determine whether it resonated with their experiences.

## Results

Several facilitators and barriers were identified that influenced implementation of sociodemographic data collection. The majority of factors that emerged originated from three of the five CFIR domains; characteristics of the intervention, inner setting and characteristics of individuals. Constructs from the outer setting and process of implementation were scarcely mentioned by study participants in any of the sites. Table 1 summarizes the various factors by site and a more detailed discussion is presented in the following sections.

### Facilitators to implementation

#### Intervention characteristics

Intervention characteristics refer to the features of sociodemographic data collection that influenced implementation. Two CFIR constructs, the relative advantage conferred by collection of sociodemographic data and the adaptability to each context, were mentioned in all cases. Participants perceived that sociodemographic data collection was beneficial and satisfied an important information need. Data generated could help managers to understand the sociodemographic profile of clients who accessed services, support advocacy for funding and inform planning for service improvement. A participant explained,

Currently we don’t have a lot of demographic data. We have very basic data on gender, age and a little bit on sexuality as well. But that doesn’t really tell us who it is that we are serving and what kinds of other needs they are experiencing. And too having that information gives us the ability to seek out funding opportunities to provide better supports and to enhance the clinical stuff that we are doing… So I think from that perspective, it is really important and I think it also enables us to tailor our services and make changes to better suit whoever it is that we are seeing to meet their needs. (K1_Case2)

#### Outer setting

The outer setting refers to influences external to the organization that drive adoption and implementation of an intervention. One CFIR construct related to external policies and incentives was mentioned in Case 2. The manager discussed the organization’s need to expand sociodemographic data collection to respond to funders who were requiring more information about the recipients of services. She said, “It is something that our funders and asking for that we are currently not collecting” (K1_Case 2).

#### Inner setting

Two related constructs of the inner setting domain that were key facilitators included relative priority accorded to implementation of sociodemographic data collection and participants’ perceived tension for change. Participants perceived that data collection was important and supported by organizational priorities. The following extract illustrates how district-level priorities served as an impetus for implementation.

Last year at district review, we identified that we wanted to focus on sociodemographic aspects of client care and really meet the needs of clients … So that was the rationale behind it because there was talk about looking more in-depth at the sociodemographic factors for clients and the proportionate universalism that we can provide to clients. (K1_Case1)

Similarly in Case 2, implementation of data collection aligned with the organization’s core service priorities and concern for social justice and equity. The following extract illustrates the prominence and organizational drive to implement collection of sociodemographic data.

It resonated with me because I really believe so strongly that the social determinants of health are a factor in our clients’ lives and that we are not necessarily addressing that in the best way possible. I think that we are health equity seeking and we really believe in social justice, eliminating poverty and helping people to be their best selves and be able to make the choices that are right for them. For me, having a belief in reproductive justice and the social determinants made me feel that this is something that was going to be challenging but was important for us to do. (K1_Case2)

Readiness for change, another construct within the inner setting domain, was important for implementation. Participants also described the importance of leadership engagement, support and commitment as key facilitators across cases. Engaged leaders approached the project team, identified intersections with their current work, and leveraged resources. The integral role of leadership support is exemplified in the following extract.

I think it was just the right time in terms of readiness for other departments to be involved and for other people to be involved and support it… [Person’s name] had a huge part in it. She looked at it from a very different lens as well because of her work in the [name of unit]…Yeah so definitely there was readiness within our department, the [name of unit] and then we had an opportunity to utilize other supports to make it happen which was really, really great. (K1_Case 1)

Another participant provided examples of leadership engagement and viewed the initiative as an indicator of the good will and receptiveness to advancing systems change and inclusiveness of First Nations and Métis peoples.

We are making big strides with the TRC [Truth and Reconciliation Commission] Calls for Action and the flag raising ceremony that occurred and big strides in our leadership. The CEO here [name of site] and the VPs have started to receive some training on cultural competence and safety awareness protocols. They are starting to appreciate the world view of First Nations and Métis peoples because we have our own ways of knowing. We have a long way to go but by the leadership acknowledging that was needed is huge towards creating that systems change. This is a small but mighty step – [name of initiative] - into understanding that because it gives that data that the people need to be empowered. (K1_Case3)

### Barriers to implementation

#### Intervention characteristics

Adaptability of the approach to implementation of sociodemographic was necessary to meet the needs of each organization; however, it also posed challenges for standardization. Support varied for collection of different sociodemographic questions across cases. For example, organizations often perceived that only a subset of questions was relevant to service delivery and this translated to reluctance to include the full complement of sociodemographic questions. A manager explained her rationale for excluding one of the sociodemographic questions.

Yeah that [sexual orientation] doesn’t affect how we provide service to clients so I didn’t feel it was necessary… For our services, it really isn’t relevant. It doesn’t matter what their sexual orientation is because we are going to provide them the same care regardless. (K1_Case1)

In Case 3, the hospital asked a single question about Indigenous identity; although managers recognized the intersection between patients’ multiple identities (e.g. ethnicity, living in poverty, housing instability). There was lower acceptability for asking about some social determinants depending on the organizational and service context.

#### Inner setting

Within the inner setting domain, organizations perceived that the availability of resources was a barrier to implementation of sociodemographic data collection. It was felt that implementation would require longer appointments to accommodate clients with complex needs***.*** Although concern partly reflected providers’ beliefs about the effects of implementation on work flow, it also revealed a larger issue about fragmented approaches to care that focus on specific services such as immunization. A nurse participant shared her willingness to collect the information if more time was allocated during the visit. She said, “If they want me to ask about social factors, I will ask but give me more time” (N3_Case1).

It was also perceived that clients would need more time to complete the intake questionnaire with additional sociodemographic questions. It was implied that the client would not perceive this extra time as value added. These concerns are reflected in the extract below.

I do think that time is also a factor for the patients as well. I feel like the longer it takes to fill out a form, the longer an appointment is or the more they have to wait for that appointment to finish, I feel that makes a huge difference. (K4_Case2)

Although the comments suggested a need for reorientation of services to accommodate data collection, it ignored the potential individual benefits from provider interventions to address health-related social factors at the point of care.

Despite leadership commitment additional staff and funding were identified as barriers in Case 2. The community-based organization, which depended on grants and donations, was already struggling to support its activities and service demand. There was also concern that asking about social determinants would raise expectations and demand for intervention that could not be met because of limited staff resources. These challenges were illustrated in the following extract.

Well I think for us one of the biggest challenges is funding. The current models funding are not great and don’t allow us to plan long term…We are sort of piece meal and constantly in the cycle of writing small grants and not being able to do things that are interesting or impactful. It is kind of what we can do with what we have in the moment. So I think that is the biggest challenge that we face. Yes people and time and money. (K1_Case2)

### Characteristics of individuals

This theme refers to the knowledge, attitudes and beliefs that individuals in an organization hold about the intervention. It was perceived to be a prominent barrier to implementation of sociodemographic data collection in two of the three study sites. Although it was agreed by care providers that understanding of client/family social context has a place in the provision of care, staff capacity and comfort with collection of the information were variable. A nurse participant stated, “I think that we know it is important but I don’t think that everyone would be comfortable with it” (N2_Case1).

Staff support and buy in were critical for successful implementation. During training sessions, registration clerks at the acute care site were apprehensive and shared their concerns that patients would be offended and reluctant to disclose their identity. Despite alluding to perceived patient discomfort as the primary reason for reluctance, it is likely that staff reactions are located within their own social experiences and the broader societal dialogue about what information should be collected to administer care. A key informant reflected on her experience in the extract below:

This project needed to be absorbed and bought into by the registration clerks because they are the ones that are asking and need to champion the questions… I think that is where I saw a lot of push back. Even during training, our own First Nation and Métis clerks were some of the biggest opponents that threw up the barrier saying ‘I don’t feel comfortable asking and this is not right. This is against what we should be doing’. To me it was a misunderstanding of what this kind of project means to the whole community. (K1_Case3)

## Discussion

The implementation of sociodemographic data collection requires transformational organizational change supported at multiple levels to build commitment needed to initiate and sustain efforts. Most facilitators and barriers operated at the level of the organization (inner setting) or individuals and could potentially be influenced by an overarching mandate for collection and use of information to support equity measurement and quality improvement. With increased advocacy, an enabling environment could be fostered by institutional imperatives that facilitate implementation of standardized data collection tools and processes that are integrated into routine work flows, appropriate education of patients and families and staff training.

Although implementation was context specific, the influential factors were similar to those identified by other studies that examined collection of sociodemographic data for health equity purposes [5, 6, 10, 16, 28–31]. The importance of an enabling environment that embodies commitment and leadership support for health equity actions has been acknowledged in several studies [32–35]. This was a common facilitator to implementation across cases.

The effect of external factors was evident in one case where funder requirements were a catalyst for implementation. Additionally, policy levers such as system level mandates for standardized sociodemographic data collection by hospitals in Massachusetts and Toronto have continued to be an impetus for work in this area and offer promise for accelerating local change [5, 36].Several studies describe multiple staff concerns with regard to sociodemographic data collection [37–40]. These included time constraints, perceived patient and staff discomfort, cost and perceived legal barriers. Health service personnel in the study shared similar concerns suggesting a need to increase staff capacity and reorient service design to support integration of sociodemographic data collection [5, 6, 36, 41].

The staff’s competence, self-efficacy and support for implementation of sociodemographic data collection is facilitated by their understanding of the underlying principles of equity, relationship between social position and health, and willingness to engage in difficult conversations about privilege, power and structural violence [42–44]. In one case where training was optimized, and staff attitudes were favorable, implementation barriers were reduced. To facilitate implementation, integration of training into staff service orientation, ongoing staff development, and inclusion of specific competencies and skills into job descriptions would create a culture of expectation that normalizes data collection efforts.

In the community-based organization, participants emphasized the importance of adequate resources including staff capacity, time and funding to support implementation. This was not prominent in the other two cases that were larger organizations with sustainable funding. This suggests that future efforts to scale up implementation will need to be adequately resourced. Although not emphasized by study participants, legacy systems with low functional interoperability precluded extensive electronic integration and fields for response options were limited when feasible. Increased capacity to adapt information systems is needed to support effective and sustainable integration of sociodemographic data collection and application in local health care settings [45].

In other sociodemographic data collection projects, engagement of relevant stakeholders has been an important part of the planning process and contributed to successful implementation [5, 6, 16]. In this study, the project team also sought to partner with stakeholders to build bridges for broader health equity work. An inclusive engagement process was designed with consultation of internal and external partners to better understand their information needs, preferences and concerns about collection and use of sociodemographic data collection.

The study explored multiple health settings; however, no rural sites were included. It is plausible that perceptions may differ among health service personnel or patients that limit application in these settings. Implementation was extremely context sensitive hence questions, mode of administration and application of information in care varied across settings. Consequently, factors may vary in relative importance depending on whether sociodemographic information will be applied individually at point of care or in aggregate at program level.

## Conclusion

System level mandates are needed to move beyond fragmented local approaches to sociodemographic data collection in urban health care settings. There is a need to explore important enablers and barriers in rural health care settings as well as understand the relative importance of factors that support clinical application of sociodemographic information. Standardization of comprehensive data collection tools that are integrated into information systems and routine work flows coupled with preparation of health service personnel are needed for implementation success.

## Additional file

Additional file 1: Better health for all:We ask because we care. Questionnaire with sociodemographic questions (DOCX 91 kb)

## Abbreviations

CFIR: Consolidated framework for implementation research
CIHI: Canadian Institute for Health Information
