# Supplementary material for: Facilitators and barriers of sociodemographic data collection in Canadian health care settings: a multisite case study evaluation
Source: Int J Equity Health. 2018 Dec 27;17:186. doi: 10.1186/s12939-018-0903-0 (PMC6307203; doi:10.1186/s12939-018-0903-0)
Supplement: Supplementary file 1 — Better health for all:We ask because we care. Questionnaire with sociodemographic questions (DOCX 91 kb) [file 12939_2018_903_MOESM1_ESM.docx]

We

**1a. How well do you speak English?** Check **ONE** only**:**

| Very well | Well | Not well | Not at all |
| --- | --- | --- | --- |

**1b. What language would you feel most comfortable communicating in with your health care providers?** Check **ONE** only:

| Arabic | English | Michif | Urdu |  |
| --- | --- | --- | --- | --- |
| Braille | Farsi | Punjabi | Vietnamese |  |
| Cantonese | French | Saulteaux | Other(specify) ____________________ |  |
| Chinese | German | Sign Language | Prefer not to answer |  |
| Cree | Hindi | Spanish |  |  |
| Déne | Lip reading | Tagalog |  |  |
| Dutch | Mandarin | Ukrainian |  |  |

**1c.** **Do you need an interpreter?** Yes  No  Prefer not to answer  Do not know

**2. What name do you go by?** __________________________________________________

**3a. Were you born in Canada?** Check **ONE** only:

| Yes | No* | Prefer not to answer | Do not know |
| --- | --- | --- | --- |

If **NO***, what year did you arrive in Canada? ______

**3b. Which option best describes your current immigration status?** Check **ONE** only**:**

| Canadian citizen | Refugee | Work Permit | Other (specify) _________________________ |
| --- | --- | --- | --- |
| Permanent  resident | Study  permit | Visitor | Prefer not to answer |
|  |  |  | Do not know |

*We are collecting social information from our patients to better understand and address your needs as well as find out who we serve. We will also use the information to plan programs and services for the community. It will only take 5 minutes to complete.*

*This information will be shared with your health care team and protected like your other health information. If used in research the information will be combined with that of other patients and no one will be able to identify any of the patients.*

***The questions are voluntary and you can choose ‘prefer not to answer’ for any question. This will not affect your care****.*

WeWe

**4a.** **Are you of indigenous background?** Check **ONE** only:

| Yes* | No* **(GO TO 4b**) | Prefer not to answer | Do not know |
| --- | --- | --- | --- |

If **YES***, please pick **ONE** of the following:

| First Nations (or Indian as defined by Indian Act) | Other(specify) _____________________ |
| --- | --- |
| Non-status Indian | Prefer not to answer |
| Métis | Do not know |
| Indigenous from outside of Canada |  |

**4b.** W**hich option best describes your racial or ethnic group?** Check **ONE** only:

| White North American (e.g. Canadian, American) | Asian – East (e.g. Chinese, Japanese,  Korean) |
| --- | --- |
| White European (e.g. English, Italian, Portuguese,  Russian) | Asian – South (e.g. Indian, Pakistani,  Sri Lankan) |
| Black North American (e.g. Canadian, American) | Asian – South East (e.g. Malaysian,  Filipino, Vietnamese) |
| Black African (e.g. Nigerian, Kenyan, Somali) | Mixed heritage (e.g. Black African and  White – North American)(Specify)  ____________________________ |
| Latin American (e.g. Argentinian, Chilean,  Salvadorean) | Other (specify)  _______________________________ |
| Middle Eastern (e.g. Egyptian, Iranian,  Lebanese) | Prefer not to answer |
|  | Do not know |

**5a. What is your religious or spiritual affiliation?** Check **ALL** that apply:

| I do not have a religious or spiritual affiliation | Sikh |
| --- | --- |
| Buddhist | Spiritual but no religious affiliation |
| Christian | Native spirituality |
| Hindu | Other(specify) ________________________ |
| Muslim | Prefer not to answer |
| Jewish | Do not know |

**5b. Please specify the denomination that you are affiliated with.**

**________________________________**

**5c. Do you want spiritual and cultural services?** YesNo

**6. Do you have any conditions that limit your activities of daily living?**

Yes*  No (**GO TO 7a**)

If **YES***, is this condition? Check **ALL** that apply:

| Chronic illness | Learning disability | Other(specify)________________ |
| --- | --- | --- |
| Drug or alcohol dependence | Mental illness | Prefer not to answer |
| Hearing impairment | Physical impairment | Do not know |
| Intellectual impairment | Visual impairment |  |

**7a. What is your gender?** Check **ONE** only:

| Female | Intersex |
| --- | --- |
| Male | Other(specify) ______________________________ |
| Transgender | Prefer not to answer |
| Two spirit | Do not know |
| **7b. What pronouns do you use?**   \| He/him \| They/them \| Prefer not to answer \| \| --- \| --- \| --- \| \| She/her \| Other(specify)____________________ \| Do not know \| | |
|  | |

**8. Which best describes your sexual orientation?** Check **ONE** only:

| Gay | Heterosexual  (straight) | Queer | Two spirit | Prefer not to  answer |
| --- | --- | --- | --- | --- |
| Lesbian | Bisexual | Questioning | Other(specify) _____________________ | Do not know |

**9. What is the highest level of education that you have ever completed?**

Check **ONE** only:

| Less than high  school | Some post-secondary  school | Trade certificate  or diploma | Prefer not to  answer |
| --- | --- | --- | --- |
| High school diploma  or equivalent | Post-secondary  completion | Graduate or professional degree | Do not know |

**10. Which of the following best describes your housing situation?** Check **ONE**

only**:**

| Home owner | Supportive housing | Shelter/hostel | Homeless |
| --- | --- | --- | --- |
| Renting | Approved home | Boarding home | Other(specify)  _____________________ |
| Staying with  family/friends | Group home | Correctional  facility | Prefer not to  answer |
|  |  |  | Do not know |

**11a. In the past month, how often did you and others worry that food would run**

**out before you got more?**

| Most of the time | Never | Do not know |
| --- | --- | --- |
| Sometimes | Prefer not answer |  |

**11b. In the past month, how often did you and others run out of food and you could not get more?**

| Most of the time | Never | Do not know |
| --- | --- | --- |
| Sometimes | Prefer not to answer |  |

**12a. What is your current monthly household income (take home pay)? C**heck **ONE** only:

| Less than $2,000 | $4,000 – $4,999 | $7,000 – $7,999 |  |
| --- | --- | --- | --- |
| $2,000 - $2,999 | $5,000 – $5,999 | $8,000 or more |  |
| $3,000 - $3,999 | $6,000 – $6,999 | Prefer not to answer |  |
|  |  | Do not know |  |

**12b. How many persons does this income support?** _______________________

Prefer not to answer  Do not know

***We thank you for taking the time to complete the questionnaire.***

***The information you provide us will help to improve the quality of care for all.***
